# Plant Proteomic Research 2.0: Trends and Perspectives

**DOI:** 10.3390/ijms20102495

**Published:** 2019-05-21

**Authors:** Setsuko Komatsu

**Affiliations:** Faculty of Environmental and Information Sciences, Fukui University of Technology, Fukui 910-8505, Japan; skomatsu@fukui-ut.ac.jp

Plants being sessile in nature are constantly exposed to environmental challenges resulting in substantial yield loss. To cope with the harsh environment, plants have developed a wide range of adaptation strategies involving morpho-anatomical, physiological, and biochemical traits [1]. In recent years, there has been phenomenal progress in the understanding of plant responses to environmental cues at the protein level. Advancements in the high-throughput “Omics” technique have revolutionized plant molecular biology research. Proteomics offers one of the best options for the functional analysis of translated regions of the genome and generates much detailed information about the intrinsic mechanisms of plant stress response. This special issue has 29 articles, which includes one review and 28 original articles on proteomic and transcriptomic studies. Various proteomic approaches are being exploited extensively for elucidating master regulatory proteins, which play key roles in stress perception and signaling. They largely involve gel-based and gel-free techniques, including both label-based and label-free protein quantification.

In this special issue, out of the 27 original proteomic publications, 21 articles use the gel-free technique, in which nine are label-free and 12 are label-based. Progress has been fueled by the advancement in mass spectrometry techniques, complemented with genome-sequence data and modern bioinformatic analysis; however, until now the two-dimensional electrophoresis based proteomic technique was used [2] as shown in six articles of this special issue. The review by Ray et al. [3] summarized the potential and limitations of the proteomic approaches and focused on *Quercus ilex* as a model species for other forest tree species. Regarding the progress of techniques in proteomics with other plant species, the research in *Q. ilex* moved from a gel-based strategy to a gel-free shotgun workflow. New directions in *Q. ilex* research leads to the identification of allergens in pollen grains/acorns and the characterization of wood materials, which are objectives clearly approached by proteomics [3]

The impact of diseases on crop production negatively reflects on sustainable food production and the overall economic health of the world. Five publications focus on biotic stress using various proteomic techniques. Khoza et al. [4] used a proteomic technique to identify *Arabidopsis* plasma-membrane associated candidate proteins in response to fungal treatment as well as those possibly interacting with the microbe-associated molecular pattern as ligands. They identified defense-related proteins and elucidated unknown signaling responses to this microbe-associated molecular pattern, including endocytosis. Furthermore, proteomic techniques were used to identify the mechanism in crops such as tomato [5], sugarcane [6], potato [7], and wheat [8] under biotic stress. Plants and pathogens are entangled in a continual arms race. Because plants have evolved dynamic defense and immune mechanisms to resist infection and enhance immunity for second wave attacks from the same or different types of pathogenic species, proteomics is a very useful technique for comprehensive analysis.

Wang et al. [9] and Gao et al. [10] performed proteomic analysis using the isobaric tag for relative and absolute quantification of castor and jojoba, respectively, under cold stress. Wang et al. [9] summarized that certain processes they identified cooperatively work together to establish the beneficial equilibrium of physiological and cellular homeostasis under cold stress. Gao et al. [10] indicated that photosynthesis suppression, cytoskeleton and cell wall adjustment, lipid metabolism/transport, reactive oxygen species scavenging, and carbohydrate metabolism were closely associated with the cold stress response. On the other hand, Inomata et al. [11] and Hao et al. [12] performed proteomics to identify the mechanisms in rice and lettuce, respectively, under high temperature. Inomata et al. [11] suggested that their results provide additional insights into carbohydrate metabolism regulation under ambient and adverse conditions. Hao et al. [12] indicated that a high temperature enhances the function of photosynthesis and auxin biosynthesis to promote the process of bolting, which is in line with the physiology and transcription levels of auxin metabolism. Furthermore, drought stress [13] and ultraviolet-B stress [14] were also used for mechanism analyses in maize and *Clematis terniflora* DC, respectively.

To facilitate the biotechnological improvement of crop productivity, genes, and proteins that control crop adaptation to a wide range of environments will need to be identified. This special issue includes many functional mechanisms of plants with nitrogen utilization [15], ammonium nutrition [16], cadmium exposure [17], nanoparticle treatment [18], and plant-derived smoke treatment [19]. Furthermore, various plants were used such as rice mutants [20], barley [21], *Morus alba* [22], pea cultivars [23], maize [24], tea [25], *Brunfelsia acuminate* [26], potato [27], and *Phalaenopsis* [28]. Due to the challenges faced in text/data mining, there is a large gap between the data available to researchers and the hundreds of published plant stress proteomic articles. PlantPReS is a valuable database for most researchers working in proteomics and plant stress areas [29].

Despite recent advancements, more emphasis needs to be given to the protein-extraction protocols, especially for proteins that are not abundant. Matsuta et al. [30] and Nishiyama et al. [31] used the mass spectrometry technique to identify heterotrimeric G γ4 and γ3 subunit proteins that are not abundant. As *RGG4*/*DEP1*/*DN1*/*qPE9-1*/*OsGGC3* mutants exhibited dwarfism, the tissues that accumulated Gγ4 corresponded to the abnormal tissues observed in *RGG4*/*DEP1*/*DN1*/*qPE9-1*/*OsGGC3* mutants [30]. On the other hand, as *RGG3/GS3/Mi/OsGGC1* mutants show the characteristic phenotype in flowers and consequently in seeds, the tissues that accumulated Gγ3 corresponded to the abnormal tissues observed in *RGG3/GS3/Mi/OsGGC1* mutants [31]. An amalgamation of diverse mass spectrometry technique, complemented with genome-sequence data and modern bioinformatics analysis, offers a powerful tool to identify and characterize novel proteins. This allows for researchers to follow temporal changes in relative protein abundances in developing/growing plant stage or under adverse environmental conditions.

Furthermore, organelle function, post-translational modifications, and protein-protein interactions, which are progress of proteomic research, provide deeper insight into protein molecular function. The major subcellular organelles and compartments in plant cells are nucleus, mitochondria, chloroplasts, endoplasmic reticulum, Golgi apparatus, vacuoles, and plasma membrane. The intracellular organelles and their interactions during stressful conditions represent the primary defense response. Subcellular proteomics has the potential to elucidate localized cellular responses and investigate communications among subcellular compartments during plant development and in response to biotic and abiotic stresses. This special issue includes the proteomic results in plasma membrane [4,30,31], chloroplast [11], and cell wall [17]. Additionally, the progress of proteomic research is understanding the post-translational modification such as phosphorylation [11,21,27].

Furthermore, proteomic data will be improved with convention regarding metabolomics and transcriptomics [32]. Although there have been significant advances over the years, a big gap still exists between the number of protein-coding genes and proteins detected with sufficient experimental evidence [33]. The guest editor hopes that proteomic data can detect the proteins with less experimental evidence and identify the missing proteins, which mainly use mass spectrometry-based experimental approaches. Although proteomic articles are independently published, the systematic collaborative network will be useful for further functional analyses in the near future. The articles in this special issue will be of general interest to proteomic researchers, plant biologists, and environmental scientists.

The guest editor hopes that this special issue will provide readers with a framework for understanding plant proteomics and insights into new research directions within this field. The guest editor thanks all of the authors for their contributions and thanks the reviewers for their critical assessments of these articles. Moreover, the guest editor renders heartiest thanks to the Assistant Editor, Ms. Chaya Zeng for giving me the opportunity to serve “Plant Proteomic Research 2.0” as guest editor.

## References

[1] Komatsu S., Hossain Z. (2017). Preface—Plant Proteomic Research. Int. J. Mol. Sci..

[2] Jorrin-Novo J.V., Komatsu S., Sanchez-Lucas R., Rodríguez de Francisco L.E. (2019). Gel electrophoresis-based plant proteomics: Past, present, and future. Happy 10th anniversary Journal of Proteomics!. J. Proteomics.

[3] Rey M., Castillejo M., Sánchez-Lucas R., Guerrero-Sanchez V., López-Hidalgo C., Romero-Rodríguez C., Valero-Galván J., Sghaier-Hammami B., Simova-Stoilova L., Echevarría-Zomeño S. (2019). Proteomics, Holm oak (*Quercus ilex* L.) and other recalcitrant and orphan forest tree species: How do they see each other?. Int. J. Mol. Sci..

[4] Khoza T., Dubery I., Piater L. (2019). Identification of candidate ergosterol-responsive proteins associated with the plasma membrane of *Arabidopsis thaliana*. Int. J. Mol. Sci..

[5] Fan K., Wang K., Chang W., Yang J., Yeh C., Cheng K., Hung S., Chen Y. (2019). Application of data-independent acquisition approach to study the proteome changes from early to later phases of tomato pathogenesis responses. Int. J. Mol. Sci..

[6] Singh P., Song Q., Singh R., Li H., Solanki M., Malviya M., Verma K., Yang L., Li Y. (2019). Proteomic analysis of the resistance mechanisms in sugarcane during *Sporisorium scitamineum* infection. Int. J. Mol. Sci..

[7] Xiao C., Gao J., Zhang Y., Wang Z., Zhang D., Chen Q., Ye X., Xu Y., Yang G., Yan L. (2019). Quantitative proteomics of potato leaves infected with phytophthora infestans provides insights into coordinated and altered protein expression during early and late disease stages. Int. J. Mol. Sci..

[8] Balakireva A., Deviatkin A., Zgoda V., Kartashov M., Zhemchuzhina N., Dzhavakhiya V., Golovin A., Zamyatnin A. (2018). Proteomics analysis reveals that caspase-like and metacaspase-like activities are dispensable for activation of proteases involved in early response to biotic stress in *Triticum aestivum* L.. Int. J. Mol. Sci..

[9] Wang X., Li M., Liu X., Zhang L., Duan Q., Zhang J. (2019). Quantitative proteomic analysis of castor (*Ricinus communis* L.) seeds during early imbibition provided novel insights into cold stress response. Int. J. Mol. Sci..

[10] Gao F., Ma P., Wu Y., Zhou Y., Zhang G. (2019). Quantitative proteomic analysis of the response to cold stress in jojoba, a tropical woody crop. Int. J. Mol. Sci..

[11] Inomata T., Baslam M., Masui T., Koshu T., Takamatsu T., Kaneko K., Pozueta-Romero J., Mitsui T. (2018). Proteomics analysis reveals non-controlled activation of photosynthesis and protein synthesis in a rice npp1 mutant under high temperature and elevated CO_2_ conditions. Int. J. Mol. Sci..

[12] Hao J., Zhang L., Li P., Sun Y., Li J., Qin X., Wang L., Qi Z., Xiao S., Han Y. (2018). Quantitative proteomics analysis of lettuce (*Lactuca sativa* L.) reveals molecular basis-associated auxin and photosynthesis with bolting induced by high temperature. Int. J. Mol. Sci..

[13] Zenda T., Liu S., Wang X., Jin H., Liu G., Duan H. (2018). Comparative proteomic and physiological analyses of two divergent maize inbred lines provide more insights into drought-stress tolerance mechanisms. Int. J. Mol. Sci..

[14] Chen X., Yang B., Huang W., Wang T., Li Y., Zhong Z., Yang L., Li S., Tian J. (2018). Comparative proteomic analysis reveals elevated capacity for photosynthesis in polyphenol oxidase expression-silenced *Clematis terniflora* DC. leaves. Int. J. Mol. Sci..

[15] Waqas M., Feng S., Amjad H., Letuma P., Zhan W., Li Z., Fang C., Arafat Y., Khan M., Tayyab M. (2018). Protein phosphatase (PP2C9) induces protein expression differentially to mediate nitrogen utilization efficiency in rice under nitrogen-deficient condition. Int. J. Mol. Sci..

[16] Coleto I., Vega-Mas I., Glauser G., González-Moro M., Marino D., Ariz I. (2019). New Insights on *Arabidopsis thaliana* root adaption to ammonium nutrition by the use of a quantitative proteomic approach. Int. J. Mol. Sci..

[17] Gutsch A., Zouaghi S., Renaut J., Cuypers A., Hausman J., Sergeant K. (2018). Changes in the proteome of medicago sativa leaves in response to long-term cadmium exposure using a cell-wall targeted approach. Int. J. Mol. Sci..

[18] Jhanzab H., Razzaq A., Bibi Y., Yasmeen F., Yamaguchi H., Hitachi K., Tsuchida K., Komatsu S. (2019). Proteomic analysis of the effect of inorganic and organic chemicals on silver nanoparticles in wheat. Int. J. Mol. Sci..

[19] Aslam M., Rehman S., Khatoon A., Jamil M., Yamaguchi H., Hitachi K., Tsuchida K., Li X., Sunohara Y., Matsumoto H. (2019). Molecular responses of maize shoot to a plant derived smoke solution. Int. J. Mol. Sci..

[20] Yang X., Meng W., Zhao M., Zhang A., Liu W., Xu Z., Wang Y., Ma J. (2019). Proteomics analysis to identify proteins and pathways associated with the novel lesion mimic mutant E40 in rice using iTRAQ-based strategy. Int. J. Mol. Sci..

[21] Ishikawa S., Barrero J., Takahashi F., Peck S., Gubler F., Shinozaki K., Umezawa T. (2019). Comparative phosphoproteomic analysis of barley embryos with different dormancy during imbibition. Int. J. Mol. Sci..

[22] Zhu W., Zhong Z., Liu S., Yang B., Komatsu S., Ge Z., Tian J. (2019). Organ-Specific Analysis of *Morus alba* using a gel-free/label-free proteomic technique. Int. J. Mol. Sci..

[23] Mamontova T., Lukasheva E., Mavropolo-Stolyarenko G., Proksch C., Bilova T., Kim A., Babakov V., Grishina T., Hoehenwarter W., Medvedev S. (2018). Proteome map of pea (*Pisum sativum* L.) embryos containing different amounts of residual chlorophylls. Int. J. Mol. Sci..

[24] Liu B., Shan X., Wu Y., Su S., Li S., Liu H., Han J., Yuan Y. (2018). iTRAQ-Based Quantitative proteomic analysis of embryogenic and non-embryogenic calli derived from a maize (*Zea mays* L.) inbred line Y423. Int. J. Mol. Sci..

[25] Dong F., Shi Y., Liu M., Fan K., Zhang Q., Ruan J. (2018). iTRAQ-based quantitative proteomics analysis reveals the mechanism underlying the weakening of carbon metabolism in chlorotic tea leaves. Int. J. Mol. Sci..

[26] Li M., Sun Y., Lu X., Debnath B., Mitra S., Qiu D. (2019). Proteomics reveal the profiles of color change in *Brunfelsia acuminata* flowers. Int. J. Mol. Sci..

[27] Bernal J., Mouzo D., López-Pedrouso M., Franco D., García L., Zapata C. (2019). The major storage protein in potato tuber is mobilized by a mechanism dependent on its phosphorylation status. Int. J. Mol. Sci..

[28] Chen C., Zeng L., Ye Q. (2018). Proteomic and biochemical changes during senescence of phalaenopsis ‘Red Dragon’ petals. Int. J. Mol. Sci..

[29] Mousavi S.A., Pouya F.M., Ghaffari M.R., Mirzaei M., Ghaffari A., Alikhani M., Ghareyazie M., Komatsu S., Haynes P.A., Salekdeh G.H. (2016). PlantPReS: A database for plant proteome response to stress. J. Proteomics.

[30] Matsuta S., Nishiyama A., Chaya G., Itoh T., Miura K., Iwasaki Y. (2018). Characterization of heterotrimeric G protein γ4 subunit in rice. Int. J. Mol. Sci..

[31] Nishiyama A., Matsuta S., Chaya G., Itoh T., Miura K., Iwasaki Y. (2018). Identification of heterotrimeric G protein γ3 subunit in rice plasma membrane. Int. J. Mol. Sci..

[32] Shi J., Zhao L., Yan B., Zhu Y., Ma H., Chen W., Ruan S. (2019). Comparative transcriptome analysis reveals the transcriptional alterations in growth- and development-related genes in sweet potato plants infected and non-infected by SPFMV, SPV2, and SPVG. Int. J. Mol. Sci..

[33] Rahiminejad M., Ledari M.T., Mirzaei M., Ghorbanzadeh Z., Kavousi K., Ghaffari M.R., Haynes P.A., Komatsu S., Salekdeh G.H. (2019). The quest for missing proteins in rice. Mol. Plant..

